# Effects of Plant Polyphenols on Obesity-Induced Inflammatory Depression: A Review

**DOI:** 10.3390/ijms27167452

**Published:** 2026-08-20

**Authors:** Yawei Xu, Yajie Zhang, Yunfei Huang, Lu Li, Mengna Shi, Chunmei Li

**Affiliations:** 1 College of Food Science and Technology, Huazhong Agricultural University, Wuhan 430070, China; xuyawei@webmail.hzau.edu.cn (Y.X.); hyfff@webmail.hzau.edu.cn (Y.H.); li1997@webmail.hzau.edu.cn (L.L.); smn@webmail.hzau.edu.cn (M.S.); 2 Academy of Chinese Medical Sciences, Henan University of Chinese Medicine, Zhengzhou 450000, China; zyjie2025@163.com; 3 Key Laboratory of Environment Correlative Food Science, Huazhong Agricultural University, Ministry of Education, Wuhan 430070, China

**Keywords:** obesity, depression, neuroinflammation, polyphenols, antidepressants

## Abstract

Epidemiological and clinical evidence indicates that obesity-associated metabolic dysfunction increases the risk of depression, which is often resistant to conventional antidepressant therapies. Elucidating targeted mechanisms underlying obesity-related depression is therefore essential for developing effective interventions. Accumulating evidence identifies neuroinflammation as a central link between obesity and depressive disorders. Obesity-driven inflammatory signaling disrupts multiple neurobiological processes implicated in depression, thereby contributing to the onset and progression of inflammatory depression. Polyphenols, a diverse class of plant-derived secondary metabolites, have demonstrated neuroprotective properties, attributed to their anti-inflammatory, antioxidant, and metabolic regulatory effects. By restoring neurobiological homeostasis, polyphenols may represent a promising therapeutic strategy for obesity-related inflammatory depression. This review summarizes the pathophysiological mechanisms through which obesity induces neuroinflammation and subsequently promotes depressive symptoms. It further evaluates current evidence regarding the efficacy and effective dosing of polyphenols in attenuating inflammatory signaling and alleviating obesity-related inflammatory depression, as well as the translational challenges faced in their clinical application. Given their anti-neuroinflammatory potential, polyphenols may serve as a valuable adjunctive approach for the management of obesity-related inflammatory depression.

## 1. Introduction

According to the World Health Organization (WHO), 2.5 billion adults (18 years and older) are overweight globally in 2022, with 890 million of them suffering from obesity. Furthermore, WHO estimates that by 2035, there will be 1.9 billion obese people globally, with 1 in 4 persons on average being obese [1].

Obesity substantially increases the risk of multiple chronic conditions, including cancer [2], diabetes [3], depression [4], respiratory syndrome [5] and cardiovascular disease [6]. In recent years, the association between obesity and depression has attracted growing attention. Meta-analyses indicate that obesity increases the risk of depression by 65% in older adults and 32% in the general adult population [7,8]. Among children and adolescents, the prevalence of depression is 1.34 times higher in those with obesity than in their normal-weight peers [9]. Clinically, obesity-related depression often presents with atypical features, such as increased appetite or weight gain, hypersomnia, leaden paralysis, and heightened sensitivity to interpersonal rejection-symptoms that differ from the appetite loss and insomnia commonly observed in melancholic depression [10]. Epidemiological and clinical studies suggest that obesity-induced depression is closely linked to a chronic low-grade inflammatory state driven by immune activation, with neuroinflammation emerging as a central mechanism [11]. Current antidepressant therapies primarily target monoamine neurotransmitters, including serotonin (5-HT), norepinephrine (NE), and dopamine (DA). However, these treatments often show limited efficacy in patients with obesity-related depression [12], suggesting that mechanisms of obesity-related depression are different from those of neurotransmitter-mediated depression. Therefore, it is particularly important to explore in depth the relationship between obesity, neuroinflammation and depression. Notably, although obesity and chronic low-grade inflammation increase the risk of depression, depressive symptoms do not occur in all individuals with obesity. This heterogeneity may arise from differences in genetic background, immune regulation, metabolic status, gut microbiota, and psychosocial factors. Metabolic flexibility, preserved insulin and leptin sensitivity, and effective inflammatory control may confer resilience, whereas excessive inflammation, hypothalamic–pituitary–adrenal (HPA) axis dysregulation, mitochondrial dysfunction, and impaired gut–brain communication may increase vulnerability. Therefore, obesity-related depression is a multifactorial disorder rather than a direct consequence of obesity alone.

Polyphenols, a diverse group of plant-derived bioactive compounds, have attracted considerable interest due to their anti-inflammatory, antioxidant, and neuroprotective properties [13]. They modulate immune cell activity, regulate the expression of pro-inflammatory cytokines, and influence multiple signaling pathways implicated in depression [14]. Although numerous reviews have examined the role of polyphenols in stress-induced depression, relatively few have focused specifically on obesity-related inflammatory depression. 

To clarify the novelty of this review, we compared it with recently published reviews on related topics. As summarized in Table 1, existing reviews predominantly address general depression, stress-induced models, or neuroinflammation, with few specifically addressing obesity as the primary driver. Most also lack a systematic summary of polyphenol dosage ranges, which is critical for translational applications. In contrast, the present review offers three distinctive contributions: (i) it systematically elucidates the mechanistic cascade from obesity-driven peripheral inflammation to neuroinflammation and subsequent depressive behaviors, with an emphasis on microglial lipid metabolism; (ii) it comprehensively evaluates the anti-neuroinflammatory mechanisms of polyphenols specifically in the context of obesity-related depression, covering NF-κB, MAPK, PI3K/Akt, and gut–brain axis pathways; and (iii) it provides, for the first time, a consolidated summary of effective dose ranges of polyphenols across in vitro and in vivo models, highlighting dose–response relationships and hormetic effects. Accordingly, this review synthesizes current evidence on the mechanistic interplay between obesity, neuroinflammation, and depression. It further evaluates the therapeutic potential and effective dosing of polyphenols in modulating inflammatory pathways and mitigating obesity-related inflammatory depression. Furthermore, the current limitations of clinical translation and future research directions are discussed to provide a more balanced understanding of the potential role of polyphenols in obesity-related inflammatory depression.

## 2. Review Methodology

The literature search strategy was developed based on the conceptual framework proposed by Preiss et al. [22] and tailored to the objectives of this narrative review. A structured literature search was conducted using PubMed, Web of Science, ScienceDirect, and Google Scholar databases to identify relevant studies published between 2015 and 2026. The search strategy combined terms related to obesity-related mood disorders, neuroinflammatory mechanisms, and polyphenol interventions. The primary search terms included: “obesity”, “depression”, “mood disorders”, “neuroinflammation”, “microglia”, “HPA axis”, “neurogenesis”, “insulin and leptin resistance”, “polyphenols”, “bioactive compounds”. Relevant studies were selected based on methodological quality, clarity of experimental evidence, and their contribution to understanding the biological mechanisms, therapeutic potential, and translational implications of polyphenols in obesity-related inflammatory depression. Since this review aimed to provide a comprehensive and multidisciplinary synthesis of emerging evidence rather than perform quantitative evaluation or meta-analysis, a formal systematic review protocol, such as PRISMA, was not applied. The identified literature was critically evaluated and thematically organized to highlight current mechanistic insights, clinical relevance, knowledge gaps, and future research directions.

## 3. Causes of Obesity-Induced Neuroinflammation

**Figure 1:**
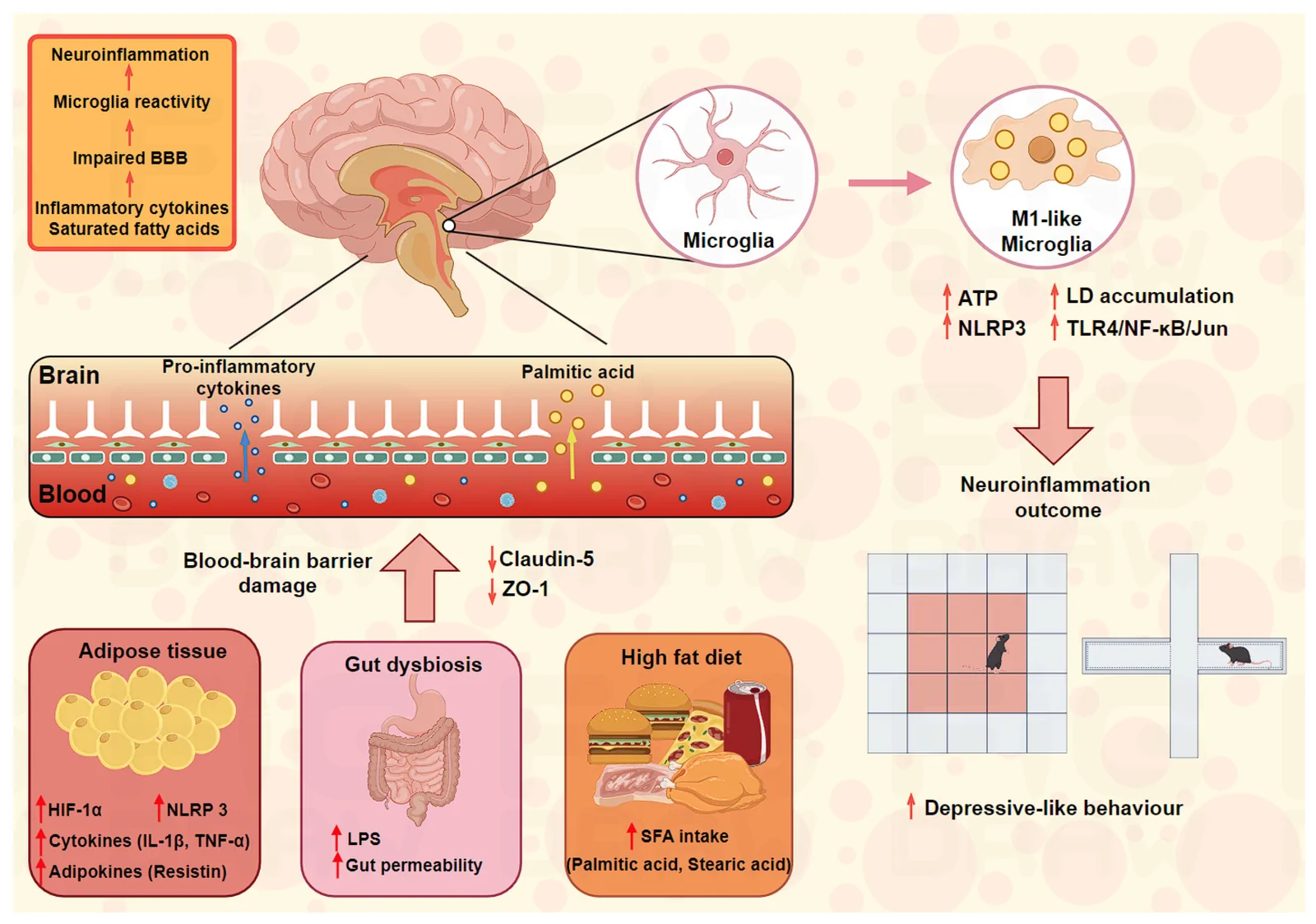
A major pathway for obesity-induced neuroinflammation. Rapid expansion of adipose tissue, altered gut microbiota, and SFA in HFD increase peripheral inflammation levels in obesity. Under the constant stimulation of inflammation, the integrity of the BBB is disrupted, which permits peripheral inflammatory cytokines and SFA to cross the BBB to activate microglia triggering neuroinflammation and depressive-like behaviours.

Microglia are resident immune cells in the central nervous system (CNS) and play an important role in neuroinflammation. Under normal physiological conditions, microglia exist in a resting state. In response to tissue injury, pathogens, or metabolic stress, they become activated and adopt an amoeboid morphology [23]. Activated microglia are broadly categorized into a pro-inflammatory (M1-like) phenotype and an anti-inflammatory or reparative (M2-like) phenotype. M1-like microglia secrete pro-inflammatory cytokines that contribute to neuronal dysfunction, whereas M2-like microglia promote debris clearance, neuronal protection, and tissue repair [24]. Current evidence linking obesity to microglial activation is derived from multiple experimental levels. Human studies have demonstrated that individuals with obesity exhibit increased neuroinflammatory markers and altered microglial activity in brain regions involved in metabolic regulation and emotional processing, including the hypothalamus and hippocampus [25,26]. However, direct evidence from human studies remains limited due to the difficulty of assessing microglial function in living individuals. Therefore, most mechanistic insights are obtained from animal models, particularly high-fat diet (HFD)-induced obesity models, which reproduce several metabolic features of human obesity. In addition, cellular inflammatory models, such as lipopolysaccharide (LPS)- or saturated fatty acid (SFA)-stimulated microglia, provide important mechanistic information but should be considered complementary rather than direct models of obesity-associated neuroinflammation. In this section, we summarize current evidence regarding how obesity-associated metabolic disturbances promote microglial activation and neuroinflammatory responses, while distinguishing direct evidence from indirect mechanistic evidence (Figure 1).

### 3.1. Peripheral Inflammation and Blood–Brain Barrier (BBB) Disruption

Obesity is characterized by chronic low-grade systemic inflammation, which represents one of the major biological pathways connecting peripheral metabolic dysfunction with CNS inflammation. Human cohort studies have demonstrated increased circulating inflammatory markers, including C-reactive protein (CRP), in individuals with obesity, and elevated inflammatory status is associated with increased risk of depressive symptoms [27]. However, whether systemic inflammation directly causes neuroinflammation in humans remains difficult to establish, and mechanistic evidence mainly comes from experimental models. Animal studies indicate that excessive adipose tissue expansion during obesity induces adipocyte hypertrophy, local hypoxia, and activation of inflammatory pathways, including hypoxia-inducible factor-1α (HIF-1α)-dependent signaling and NOD-like receptor thermal protein domain associated protein 3 (NLRP3) inflammasome activation [28]. These alterations promote secretion of pro-inflammatory cytokines and contribute to systemic inflammatory responses [29]. In addition, obesity-associated gut microbiota dysbiosis may increase circulating bacterial components, such as LPS, thereby further enhancing metabolic inflammation [4]. Peripheral inflammatory signals may influence CNS function through disruption of BBB integrity. Experimental studies demonstrate that inflammatory cytokines can reduce the expression of tight junction proteins, including claudin-5 and zonula occludens-1, resulting in increased BBB permeability [30,31]. Moreover, LPS-based experimental studies have shown that inflammatory stimulation can impair endothelial function through inhibition of P-glycoprotein activity, induction of matrix metalloproteinases, and promotion of oxidative stress-related cellular damage [32]. Although these LPS studies do not directly represent obesity, they provide mechanistic evidence explaining how inflammatory mediators may compromise BBB integrity. Following BBB disruption, peripheral cytokines may enter the CNS and influence microglial activity. Experimental evidence suggests that cytokines such as IL-1β can stimulate ATP release from microglia, promoting Ca^2+^-dependent NLRP3 inflammasome activation and subsequent inflammatory cytokine production [33]. In addition, obesity-related metabolic factors, including SFAs and adipokines such as resistin, may activate microglial Toll-like receptor 4 (TLR4) signaling, thereby amplifying inflammatory responses [34,35]. Collectively, human and animal evidence supports a model in which obesity-associated peripheral inflammation contributes to neuroinflammatory activation, although the precise causal relationship in humans requires further clinical investigation.

### 3.2. Dietary Saturated Fatty Acids (SFAs) as Triggers of Microglial Activation

High-energy diets enriched in SFAs represent a major contributor to obesity development and metabolic inflammation. Epidemiological studies indicate that increased consumption of saturated fat and elevated circulating palmitic acid (PA) levels are associated with higher inflammatory markers and depressive symptoms [36,37]. In HFD-induced obesity models, increased dietary SFA intake promotes lipid accumulation and inflammatory activation in the brain. Zhuang et al. [38] demonstrated that HFD feeding increased PA deposition in the hippocampus of mice and was accompanied by microglial activation, as indicated by altered microglial morphology and increased CD 68 expression. These findings provide direct evidence linking obesity-related dietary patterns with microglial dysfunction. Complementary in vitro studies using PA-or stearic acid-treated microglia have revealed that SFAs can activate inflammatory signaling pathways, including TLR4/NF-κB and c-Jun pathways, leading to increased production of pro-inflammatory cytokines and impaired neurogenic processes [39,40]. Although these cellular models do not reproduce the systemic metabolic alterations observed in obesity, they provide mechanistic evidence supporting the role of lipid-derived inflammatory signaling in microglial activation.

### 3.3. Lipid Droplet (LD) Accumulation in Microglia

Recent advances in immunometabolism have highlighted LD accumulation as a potential mechanism linking metabolic stress with persistent neuroinflammatory activation. Traditionally considered passive lipid storage organelles, LDs are now recognized as dynamic intracellular structures involved in lipid trafficking, inflammatory signaling, oxidative stress regulation, and cellular adaptation to metabolic challenges [41]. In peripheral immune cells, excessive LD accumulation is associated with enhanced inflammatory responses and impaired immune function [42]. Emerging evidence suggests that similar metabolic reprogramming occurs in microglia, indicating that LD dysregulation may represent an important molecular bridge between obesity-associated metabolic abnormalities and neuroinflammation. Direct evidence supporting the involvement of microglial LD accumulation in obesity-associated neuroinflammation mainly comes from HFD-induced obesity models. Prolonged HFD exposure promotes excessive lipid deposition in the brain and induces LD accumulation in hippocampal microglia, which is accompanied by altered emotional behaviors and increased microglial activation markers in mice [38,43,44]. These findings suggest that obesity-related lipid overload may drive microglial metabolic adaptation and contribute to neuroinflammatory alterations associated with mood disturbances. However, because evidence from human studies remains limited, whether microglial LD accumulation directly occurs in individuals with obesity-related depressive symptoms requires further investigation. Mechanistic studies using inflammatory and metabolic stress models provide additional insights into how LDs regulate microglial inflammatory function. Although these models do not fully reproduce obesity-associated neuroinflammation, they help identify molecular pathways involved in LD formation and inflammatory amplification. For example, activation of innate immune signaling through TLR4 has been shown to stimulate de novo LD synthesis in microglia following inflammatory stimulation [45,46]. Similarly, lipid-laden microglia have been identified in aging-related neurodegenerative conditions and are characterized by impaired lipid metabolism, increased oxidative stress, and a pro-inflammatory phenotype [46]. Together, these findings indicate that LD accumulation may represent a conserved response of microglia to chronic metabolic or inflammatory stress.

## 4. Pathways of Neuroinflammation Triggering Depression

**Figure 2:**
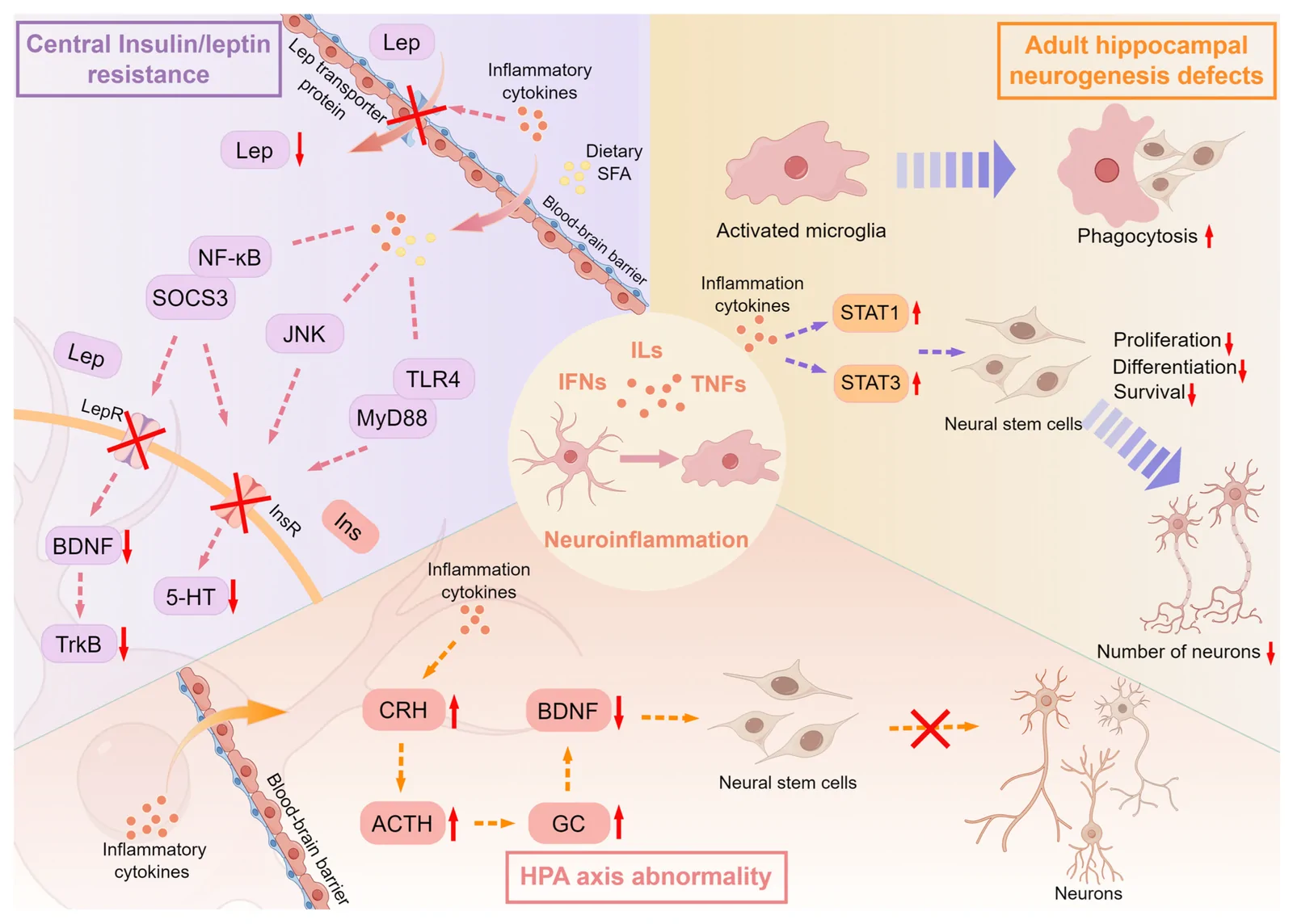
Mechanisms implicated in obesity-induced inflammatory depression. (i) Abnormalities of the HPA axis; (ii) insulin and leptin resistance in the hypothalamus and hippocampus; (iii) defects in adult hippocampal neurogenesis. Each of these mechanisms has been tied to obesity or poor diet-induced neuroinflammation.

Increasing evidence suggests that obesity-associated neuroinflammation represents an important biological interface linking metabolic disturbances to depressive phenotypes. In this section, we discuss the major neurobiological pathways through which neuroinflammation may contribute to depressive phenotypes, including HPA axis dysregulation, impaired adult hippocampal neurogenesis, and disruption of central insulin and leptin signaling (Figure 2).

### 4.1. Abnormalities of the HPA Axis

The HPA axis represents a major neuroendocrine system-regulating stress response and has been implicated in both obesity and depression. Evidence from clinical studies indicates that individuals with obesity frequently exhibit altered HPA axis activity, including increased cortisol secretion and impaired glucocorticoid feedback regulation [47,48]. Mechanistic studies suggest that obesity-related inflammatory activation may influence HPA axis function through cytokine-mediated signaling. Activated microglia and peripheral immune cells release pro-inflammatory cytokines, among which IL-1β has been proposed as an important regulator of HPA axis activation. IL-1β can stimulate hypothalamic corticotropin-releasing hormone (CRH) secretion and subsequently increase pituitary adrenocorticotropic hormone (ACTH) release, leading to elevated glucocorticoid production, including cortisol in humans and corticosterone in rodents [19,49]. Notably, the relationship between the HPA axis and obesity appears to be bidirectional. Elevated glucocorticoid levels may promote obesity development by increasing appetite, enhancing visceral adipogenesis, and reducing energy expenditure through impaired brown adipose tissue thermogenesis. Conversely, obesity-associated inflammatory stress may further activate the HPA axis, potentially establishing a vicious cycle that increases vulnerability to mood disturbances [50]. Animal studies provide mechanistic evidence linking glucocorticoid signaling with hippocampal dysfunction. In db/db mice, increased corticosterone suppresses hippocampal brain-derived neurotrophic factor (BDNF) expression via activation of glucocorticoid receptors [51]. In contrast, lentiviral suppression of the glucocorticoid receptor expression restores BDNF expression and rescues hippocampal neurogenesis in db/db mice. Therefore, obesity-related depression may be associated with impaired hippocampal neurogenesis induced by elevated corticosterone.

### 4.2. Defects in Adult Hippocampal Neurogenesis

Adult hippocampal neurogenesis is considered an important biological process involved in mood regulation and cognitive function, and accumulating evidence suggests that obesity-associated inflammation may contribute to this impairment. Direct evidence from HFD-induced obesity models demonstrates that chronic dietary metabolic stress reduces hippocampal neurogenesis. HFD-fed mice exhibit decreased numbers of doublecortin (DCX)-positive immature neurons and Ki67-positive proliferating cells in the dentate gyrus, accompanied by depressive-like behavioral alterations, including reduced sucrose preference and altered exploratory activity [52]. These findings provide relatively strong evidence linking obesity-associated metabolic disturbance with hippocampal structural and behavioral abnormalities. Inflammatory mechanisms underlying impaired neurogenesis have been further explored using cellular and inflammatory models. Pro-inflammatory cytokines, including IL-6, IL-1β, and TNF-α, can suppress neural progenitor cell proliferation through activation of STAT3 signaling, whereas interferon-related pathways inhibit neurogenesis through STAT1 activation [53,54,55,56]. Although these studies mainly use inflammatory stimulation models, they provide mechanistic explanations for how obesity-associated cytokine elevation may influence hippocampal function. Microglial dysfunction represents another potential mechanism regulating neurogenesis. In LPS-induced inflammatory models, pharmacological inhibition of microglial activation restores hippocampal neurogenesis, suggesting that excessive inflammatory microglial responses negatively affect newborn neuron survival [57]. Importantly, HFD-fed mice also show enhanced microglial phagocytosis of immature neurons, which contributes to impaired neurogenesis and depressive-like behaviors [52]. Treatment with anti-inflammatory agents such as minocycline reduces microglial activation and partially restores neurogenesis [58].

### 4.3. Resistance of Central Insulin and Leptin

Disruption of central metabolic signaling represents another potential pathway connecting obesity-associated inflammation with depressive symptoms. Insulin and leptin are important metabolic hormones that regulate energy homeostasis, neuronal plasticity, and emotional behaviors. Epidemiological studies have demonstrated that reduced insulin sensitivity is associated with increased depressive and anxiety symptoms in individuals with obesity [59]. Animal studies provide additional evidence that obesity-related metabolic dysfunction affects brain function. HFD-induced insulin resistance in rodents is accompanied by anxiety- and depression-like behaviors [60]. Furthermore, selective reduction of insulin receptor expression in astrocytes promotes depression-like behaviors in mice, suggesting that central insulin signaling contributes to mood regulation [61]. Inflammatory signaling appears to be a major mediator of central insulin resistance. Experimental studies demonstrate that inflammatory cytokines impair insulin receptor signaling through activation of IKKβ/NF-κB, JNK, and TLR4 pathways [34,62]. In HFD models, dietary SFAs activate TLR4/MyD88 signaling and enhance endoplasmic reticulum stress, contributing to hypothalamic insulin resistance [63]. Although these findings are mainly derived from animal studies, they provide mechanistic evidence linking obesity-related inflammation with impaired neuronal metabolic regulation. Leptin signaling is similarly affected by obesity-associated inflammation. Under physiological conditions, leptin acts through leptin receptors (LepR) in the hypothalamus and hippocampus to regulate energy balance and exert neuroprotective effects. However, obesity-induced inflammation may impair leptin transport across the BBB by reducing leptin transporter expression, thereby limiting central leptin availability [64]. In addition, impaired hippocampal leptin receptor signaling reduces the antidepressant-like effects of leptin administration in diet-induced obese mice [65]. Overall, current evidence suggests that obesity-associated inflammation may disrupt central insulin and leptin signaling, thereby impairing neuronal plasticity and increasing susceptibility to depressive phenotypes.

## 5. Potential Mechanisms of Polyphenols Ameliorating Obesity-Related Inflammatory Depression

**Figure 3:**
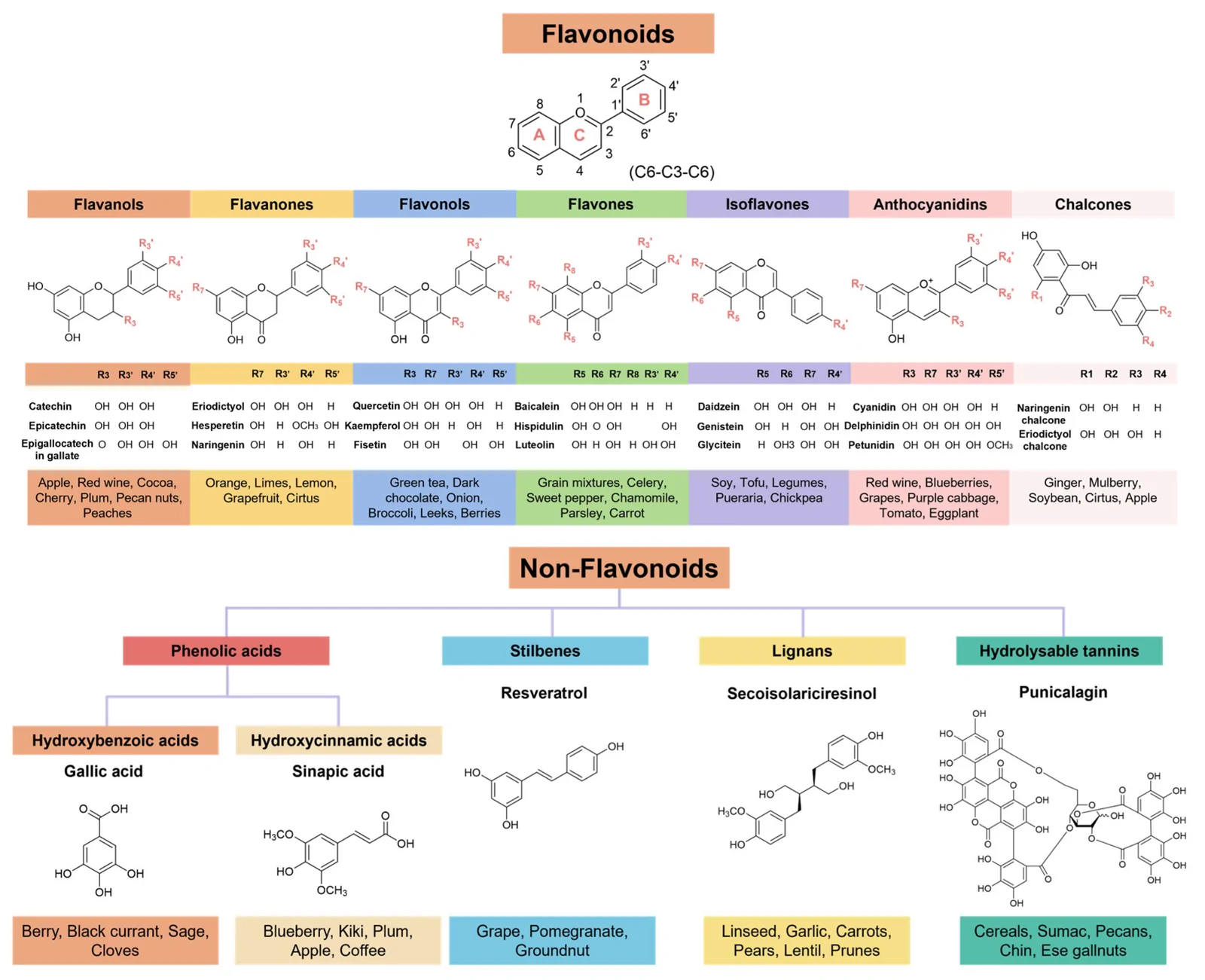
Classification, chemical structure and main sources of polyphenols.

Polyphenols are a diverse group of plant-derived secondary metabolites characterized by aromatic structures containing one or more hydroxyl groups [66]. According to their chemical structures, polyphenols are generally classified into flavonoids and non-flavonoids (Figure 3). Flavonoids possess a C6-C3-C6 skeleton and include flavanols, flavones, flavonols, anthocyanidins, flavanones, isoflavones, and chalcones [67]. Non-flavonoid polyphenols mainly consist of phenolic acids, stilbenes, lignans, and hydrolysable tannins, among which phenolic acids represent the major subclass [68].

**Figure 4:**
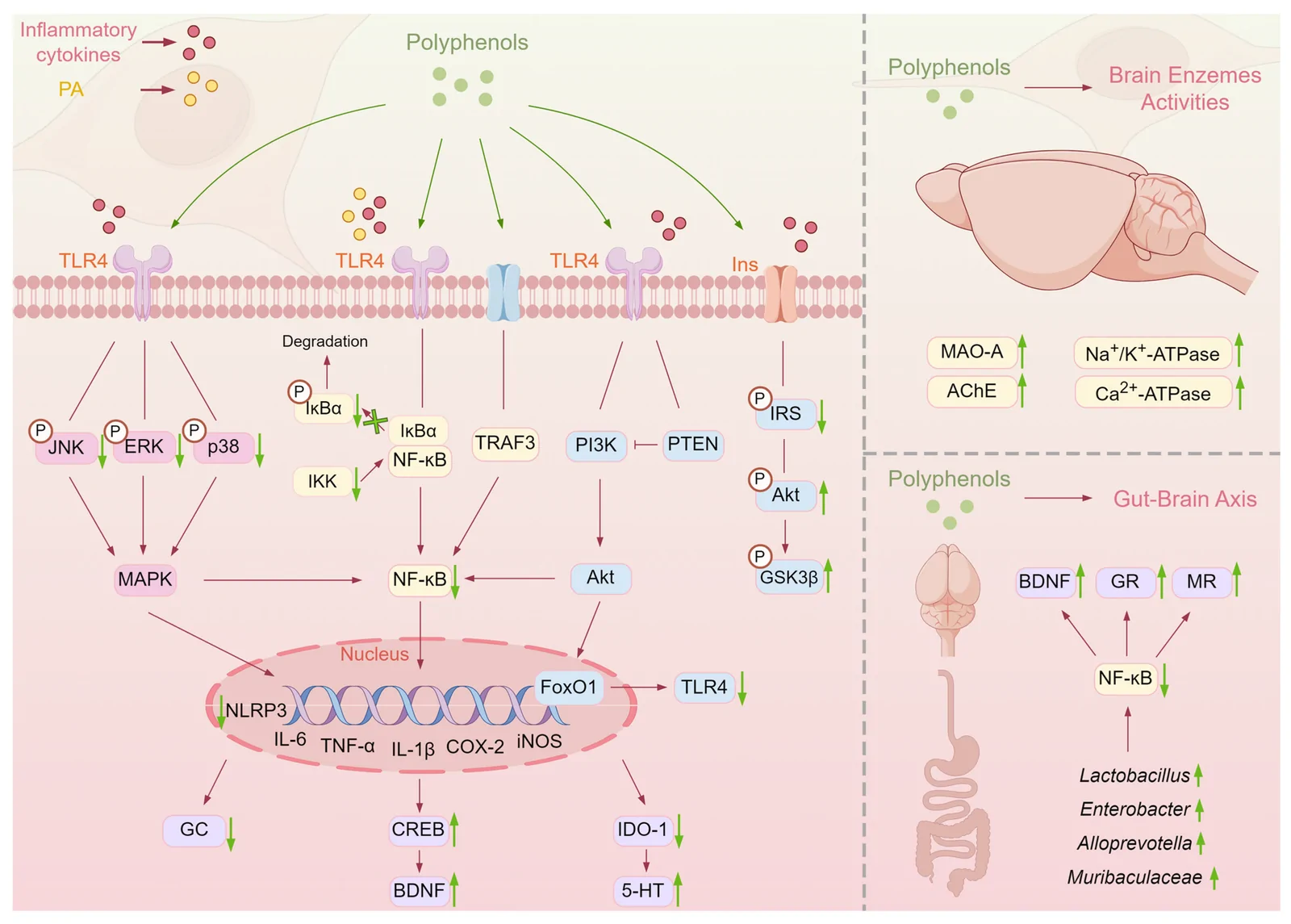
Summary of potential anti-neuroinflammatory mechanisms implicated in the antidepressant-like effects of polyphenols in obesity and neuroinflammation-induced depression models. Polyphenols can inhibit neuroinflammatory responses via the NF-κB pathway, MAPK pathway, and PI3K/Akt pathway, and ultimately alleviate associated depressive symptoms by modulating HPA axis activity, BDNF expression, and 5-HT synthesis. In addition, polyphenols can exert anti-neuroinflammatory and antidepressant effects by regulating the gut–brain axis and brain enzymes’ activities.

Accumulating evidence indicates that polyphenols exhibit anti-inflammatory, antioxidant, metabolic regulatory, and neuroprotective properties, suggesting their potential role in regulating obesity-related mood disorders [69]. The biological effects of polyphenols have been investigated using diverse experimental approaches. Obesity-related models, particularly HFD-induced models, provide disease-relevant evidence by reproducing metabolic abnormalities associated with obesity, whereas inflammatory stimulation models and cellular systems provide mechanistic insights into specific molecular pathways targeted by polyphenols. Integration of evidence from different experimental systems is therefore important for understanding how polyphenols may influence obesity-associated inflammatory depressive phenotypes. The subsequent sections explore how phenolic compounds alleviate inflammatory depression via the specific signaling pathways (Figure 4).

### 5.1. Modulation of Hippocampal NF-ĸB Pathway

The NF-κB signaling pathway represents a central regulator of inflammatory responses and plays an important role in obesity-associated neuroinflammation. Evidence from HFD-induced obesity models demonstrates that chronic dietary fat overload activates hippocampal NF-κB signaling, characterized by increased phosphorylation of IKKα/β, IκBα, and p65, which is accompanied by depression-like behaviors in rodents [70]. These findings support the involvement of NF-κB-mediated neuroinflammation in obesity-related mood disturbances. Polyphenols have been shown to counteract NF-κB activation in obesity-related models. For example, apple polyphenols suppress hippocampal NF-κB-related inflammatory responses, reduce circulating corticosterone and adrenocorticotropic hormone levels, restore BDNF expression, and improve depressive-like behaviors in HFD-fed mice [71]. This study provides direct evidence supporting the potential involvement of NF-κB inhibition in the beneficial effects of polyphenols under obesity-associated conditions. Complementary mechanistic studies using inflammatory stimulation models further demonstrate that polyphenols can regulate NF-κB activation in microglia. LPS, TNF-α, and PA induce microglial inflammatory responses through NF-κB activation [72,73], whereas polyphenols such as baicalein, salidroside, and fisetin suppress NF-κB-related inflammatory signaling and restore neuroprotective pathways [74,75,76]. These findings provide mechanistic support that NF-κB represents a conserved molecular target of polyphenols. However, further studies using obesity-related depression models are required to determine whether these mechanisms directly contribute to behavioral improvements.

### 5.2. Modulation of MAPK Pathway in CNS

Mitogen-activated protein kinase (MAPK) pathways, including ERK1/2, p38, JNK, and ERK5, integrate inflammatory and stress-related signals involved in neuroimmune activation and neuronal plasticity [16]. HFD and high-fat/high-fructose diet (HFFD) feeding increase JNK and p38 activation, contributing to neuroinflammation and cognitive dysfunction in rodents [35,77]. These observations suggest that MAPK activation represents an important mechanism linking obesity-associated metabolic disturbances with CNS dysfunction. Polyphenols may regulate MAPK signaling through inhibition of excessive phosphorylation cascades. Several flavonoids and phenolic compounds, including quercitrin, baicalin, sinapic acid, and sophoraflavanone G, suppress phosphorylation of JNK, p38, and ERK, thereby reducing inflammatory responses [78,79,80,81]. In obesity-related models, isorhamnetin attenuates HFFD-induced neuroinflammation through inhibition of JNK, p38, and NF-κB pathways, whereas punicalagin suppresses MAPK activation and inflammatory responses [82,83]. Additional evidence from LPS-activated microglial models demonstrates that fisetin selectively inhibits ERK phosphorylation and reduces inflammatory mediators, including iNOS and COX-2 [84]. These studies indicate that polyphenols may exert anti-inflammatory effects through selective regulation of MAPK components.

### 5.3. Modulation of Hippocampal PI3K/Akt Pathway

The phosphatidylinositol-3-kinase (PI3K)/protein kinase B (Akt) pathway functions as an important mediator linking metabolic homeostasis, inflammation, and neuronal survival. Under physiological conditions, insulin receptor activation stimulates IRS/PI3K/Akt signaling, promoting neuronal plasticity and survival [85]. In obesity, impaired insulin sensitivity disrupts this pathway and contributes to metabolic dysfunction associated with increased vulnerability to depressive symptoms [86]. Several studies using obesity-related metabolic models demonstrate that polyphenols can restore impaired PI3K/Akt signaling. Genistein and ferulic acid improve neuronal function in obese diabetic animals by regulating IRS, Akt, and GSK3β phosphorylation [87,88]. Similarly, EGCG and sea-buckthorn flavonoids alleviate HFFD-induced insulin resistance and neuronal injury by activating IRS/PI3K/Akt and ERK/CREB/BDNF pathways [89,90]. Mechanistic studies further reveal interactions between PI3K/Akt signaling and inflammatory pathways. In LPS-induced inflammatory models, activation of PI3K/Akt/NF-κB signaling contributes to neuroinflammatory responses, whereas curcumin, EGCG, and eriodictyol suppress inflammatory cytokine production by modulating this pathway [91,92,93]. These findings suggest that PI3K/Akt represents a potential target through which polyphenols may coordinate metabolic and inflammatory regulation.

### 5.4. Modulation of Other Pathways

In addition to central inflammatory pathways, increasing evidence suggests that polyphenols may influence obesity-related depressive phenotypes through modulation of the gut–brain axis. HFD-induced gut microbiota dysbiosis contributes to systemic inflammation, altered microbial metabolite production, and behavioral abnormalities, providing a potential target for nutritional interventions [94]. EGCG has been reported to improve HFD-induced gut microbiota imbalance and depressive-like behaviors by increasing beneficial bacterial populations, including *Muribaculaceae* and *Alloprevotella*, thereby reducing intestinal inflammation and regulating microbial-derived signals involved in CNS function [95]. Similarly, (-)-epicatechin improves HFD-associated behavioral abnormalities by modifying gut microbial composition, particularly increasing *Lactobacillus* and *Enterobacter* abundance [96]. These findings support the potential involvement of microbiota-mediated mechanisms, although causal relationships between microbial alterations and behavioral improvement require further investigation. Polyphenols may also regulate neurotransmitter metabolism and neuronal enzyme activity. Blueberry polyphenol extracts reduce LPS-induced neuroinflammation and depressive-like behaviors by modulating monoamine oxidase-A (MAO-A), acetylcholinesterase (AChE), and Na^+^/K^+^-ATPase activities [97]. Tannic acid similarly exhibits neuroprotective effects through regulation of ATPase activity [98]. However, these findings are primarily derived from inflammatory models, and whether similar mechanisms occur in obesity-related depressive conditions remains unclear.

## 6. Experimental Dosage Considerations of Polyphenols in Ameliorating Obesity-Related Inflammatory Depression

Although numerous studies have demonstrated the anti-inflammatory, antioxidant, and neuroprotective properties of polyphenols, the optimal dosage required to regulate obesity-related depressive phenotypes remains undetermined. Dose evaluation is important for understanding the pharmacological properties of polyphenols; however, interpretation of dosage data requires careful consideration of experimental context. Currently, reported doses are derived from heterogeneous experimental systems, including cellular models, animal studies, different polyphenol preparations (purified compounds versus extracts), administration routes, intervention durations, and diverse outcome measurements. Therefore, these doses should be regarded as model-specific experimental effective ranges rather than clinically validated therapeutic doses.

In this section, we summarize the reported dosage ranges of polyphenols in different experimental systems (Table 2) and discuss the factors influencing dose–response relationships, including chemical structure, bioavailability, model characteristics, and intervention strategies. Importantly, no validated dosage has currently been established for the treatment of obesity-related depression in humans.

### 6.1. Dosage Considerations in Cellular Models

Cellular models are frequently used to investigate the molecular mechanisms underlying the anti-inflammatory effects of polyphenols, particularly through stimulation of microglia with inflammatory mediators such as LPS or PA. These models allow precise evaluation of concentration-dependent effects; however, the concentrations applied in vitro cannot be directly extrapolated to physiological exposure levels in vivo. As summarized in Table 2, the effective concentrations of polyphenols in cellular models vary considerably depending on compound structure, experimental conditions, and measured outcomes. For example, baicalin and its aglycone baicalein exhibit different inhibitory capacities against LPS-induced inflammatory responses in BV2 microglia. Baicalin significantly reduces IL-1β production at 22.5 μM, whereas baicalein requires a higher concentration (45 μM) to achieve comparable effects [80,99]. These differences may be associated with variations in molecular structure, cellular uptake, metabolism, and intracellular target interaction. Compared with purified compounds, polyphenol extracts generally require higher concentrations to achieve comparable anti-inflammatory effects. For example, blueberry polyphenol extracts exhibit protective effects in microglial inflammatory models at relatively higher concentrations, which may be related to their complex composition and variable content of bioactive components [109]. Therefore, direct comparison between extract-based preparations and purified polyphenols should be interpreted cautiously. Regarding dose–response relationships, many studies report concentration-dependent inhibition of inflammatory mediators. Sinapic acid, for example, progressively reduces iNOS and IL-6 expression within the concentration range of 5–20 μM in activated microglia [79]. However, polyphenol effects are not always linearly associated with concentration. Some compounds exhibit hormetic characteristics, showing protective effects at moderate concentrations but reduced cell viability at excessive concentrations. EGCG decreases BV2 microglial proliferation at concentrations of 175–300 μM, while hesperetin induces cytotoxicity at 200 μM [73,93]. These findings highlight the importance of defining safety windows when interpreting in vitro dosage data.

### 6.2. Dosage Considerations in Animal Models

Animal studies provide more disease-relevant information regarding the effects of polyphenols on obesity-associated metabolic dysfunction and depression-like behaviors. Nevertheless, reported doses vary substantially because of differences in animal species, dietary models, administration methods, treatment duration, and evaluated biological outcomes. Among available studies, HFD-induced obesity models represent the most relevant experimental system for investigating obesity-related inflammatory depressive phenotypes. In these models, polyphenols have been administered as purified compounds or plant-derived extracts at various doses. However, these doses should not be interpreted as universal effective doses because each dosage reflects a specific experimental context. Generally, plant extracts containing multiple bioactive constituents are administered at higher doses than purified compounds. For example, apple polyphenol extracts and sea-buckthorn flavonoid preparations have been reported to exert beneficial effects at doses ranging from approximately 120 to 500 mg/kg/day [71,90]. In contrast, purified compounds such as EGCG, epicatechin (EC), and hydroxytyrosol (HT) are often evaluated at lower doses, commonly within the range of 20–100 mg/kg/day [96,108]. However, these differences primarily reflect variations in chemical composition, absorption efficiency, and experimental design rather than differences in intrinsic potency. Dose-dependent effects have been observed in several obesity-related models. For example, in HFD-fed mice, EC administration at 20 mg/kg produced greater improvements in behavioral performance, hippocampal BDNF expression, and glucocorticoid receptor regulation compared with 2 mg/kg [96]. Similarly, HT at 100 mg/kg showed stronger effects than 25 mg/kg in improving cognitive function and reducing neuroinflammatory responses [108]. Administration route and intervention duration are additional factors influencing observed dosage effects. Most animal studies employ oral administration, with intervention periods ranging from several weeks to several months depending on the obesity model. For instance, apple polyphenol extract administered at 500 mg/kg/day for 8 weeks improves depressive-like behaviors in high-sucrose diet-induced obese mice [71]. Sea-buckthorn flavonoids require longer intervention (14 weeks) to improve cognitive impairment induced by HFFD feeding [90]. These differences indicate that dosage interpretation should consider both dose intensity and exposure duration. Furthermore, preventive and therapeutic interventions may require different dosage strategies. Preventive administration before or during metabolic stress exposure may achieve benefits with relatively lower doses, whereas therapeutic intervention after establishment of obesity-associated inflammation may require longer treatment duration or higher exposure. However, systematic comparisons between preventive and therapeutic dosage strategies remain limited.

## 7. Translational Challenges of Polyphenols for Obesity-Related Inflammatory Depression

### 7.1. Limitations of Current Preclinical Evidence and Species Translation

A major challenge in translating polyphenol-based interventions from experimental models to humans is the substantial difference between animal models and human pathophysiology. Rodent models, particularly HFD-induced obesity models, are widely used to investigate the relationship between metabolic dysfunction, neuroinflammation, and depression-like behaviors. These models successfully reproduce several characteristics of human obesity, including weight gain, insulin resistance, systemic inflammation, and microglial activation [110,111]. However, they cannot fully capture the complexity of human obesity-related depression, which involves genetic susceptibility, psychosocial stress, lifestyle factors, immune regulation, and long-term metabolic alterations. Furthermore, behavioral paradigms used in rodents, such as forced swimming test, tail suspension test, and sucrose preference test, represent depression-like phenotypes rather than clinical depression itself. Therefore, improvements observed following polyphenol intervention in animal models should not be directly interpreted as antidepressant efficacy in humans. Future studies require integration of human observational studies, randomized controlled trials, and biomarker-based stratification approaches to determine whether polyphenols can exert clinically meaningful effects in individuals with obesity-related depression.

### 7.2. Bioavailability and Pharmacokinetic Limitations of Polyphenols

Poor bioavailability represents one of the major obstacles limiting the clinical application of many polyphenols. Despite strong biological activities observed in vitro, numerous polyphenolic compounds exhibit low systemic exposure due to limited intestinal absorption, extensive first-pass metabolism, rapid elimination, and chemical instability. For example, EGCG, one of the most extensively studied tea polyphenols, undergoes extensive metabolism through glucuronidation, sulfation, and methylation, resulting in relatively low circulating concentrations of intact EGCG after oral administration [112]. Similarly, quercetin exhibits poor aqueous solubility and extensive metabolism, with most circulating forms existing as conjugated metabolites rather than free aglycones [113]. Curcumin represents another typical example, showing rapid degradation, poor absorption, and low plasma concentrations despite potent anti-inflammatory activity observed in experimental systems [114]. These pharmacokinetic limitations create a substantial gap between concentrations used in cellular experiments and achievable concentrations in human tissues. For instance, many in vitro studies employ micromolar concentrations of polyphenols, whereas plasma concentrations after dietary intake are often considerably lower [115]. Therefore, the biological relevance of in vitro findings requires careful interpretation, and pharmacokinetic evaluation should be incorporated into future studies.

### 7.3. BBB Penetration and CNS Availability

Because obesity-related depression involves neuroinflammation within the CNS, the ability of polyphenols to reach brain tissues represents a critical determinant of therapeutic potential. However, many polyphenols exhibit limited brain bioavailability due to poor lipophilicity, rapid metabolism, and active efflux transport mechanisms at the BBB. Studies investigating CNS distribution have shown that only a fraction of orally administered polyphenols or their metabolites can reach the brain. For example, a study employing ^14^C-labeled grape polyphenols demonstrated that residual labeled material in the brain 24 h after oral administration ranged from 0.1% to 1.7% of the administered dose, and its neuroprotective effects may depend partly on peripheral mechanisms, including modulation of systemic inflammation and gut microbiota [116]. A polyphenol mixture derived from the Mediterranean diet also show restricted CNS exposure, although their metabolites may contribute to biological activity [117]. BBB permeability may not only depend on chemical structure but may also be influenced by obesity-associated pathological changes. Chronic inflammation, oxidative stress, and altered tight junction integrity during obesity may modify BBB function and potentially affect polyphenol transport [118]. However, whether obesity-induced BBB dysfunction enhances or restricts brain exposure of specific polyphenols remains insufficiently understood.

### 7.4. Role of Gut Microbiota in Modifying Polyphenol Bioactivity

Increasing evidence indicates that the biological effects of polyphenols are closely associated with gut microbiota metabolism. Many polyphenols undergo extensive microbial transformation into smaller phenolic metabolites, which may possess higher absorption efficiency and distinct biological activities compared with their parent compounds. For example, ellagitannins are metabolized by intestinal bacteria into urolithins, which exhibit anti-inflammatory and mitochondrial regulatory activities [119]. Similarly, microbial metabolites of flavonoids may contribute to their systemic and neuroprotective effects [120]. Conversely, polyphenols can reshape gut microbial composition by increasing beneficial bacteria and reducing inflammation-associated microbial populations, thereby influencing the gut–brain axis. However, interindividual variability in gut microbiota composition represents an important challenge for clinical translation. Differences in microbial metabolic capacity may result in substantial variation in polyphenol exposure and biological responses among individuals. Therefore, future precision nutrition strategies should consider microbiota characteristics when evaluating polyphenol efficacy.

## 8. Conclusion and Future Perspectives

With the rapid socio-economic development and the drastic evolution of dietary patterns, overweight, obesity and related metabolic problems caused by unbalanced diets have emerged. The incidence of depression triggered by obesity is also increasing annually. Due to the lack of response to conventional antidepressants in obese depressed patients, exploring a more targeted approach to ameliorate obesity-related depression is particularly important. This review elucidated the pathophysiological mechanisms by which obesity triggers neuroinflammation and thus depression. Meanwhile, we summarized the findings from both in vivo and in vitro studies on the ameliorative effects of polyphenols against obesity-related depression, as well as their effective dosage ranges. It should be noted that the antidepressant potential of polyphenols is not confined to the obesity-related context-in non-obesity-induced depression models, polyphenols have also been demonstrated to exert antidepressant-like effects through multiple pathways, including regulation of oxidative stress, neurotransmitter homeostasis, HPA axis activity, and neuroplasticity. However, most available evidence remains derived from cellular and animal models, and the clinical efficacy of polyphenols in individuals with obesity-associated depression remains insufficiently established.

Future research should move beyond evaluating polyphenols as general anti-inflammatory compounds and focus on identifying their precise molecular targets and responder populations. Multi-omics approaches integrating transcriptomics, lipidomics, metabolomics, and microbiome profiling may help uncover metabolic signatures associated with polyphenol responsiveness and facilitate biomarker-guided precision nutrition strategies. In particular, further investigation into microglial metabolic reprogramming, lipid droplet dynamics, and neuroimmune interactions may provide new mechanistic insights into obesity-related inflammatory depression. Moreover, improving the bioavailability and brain delivery efficiency of polyphenols represents an essential direction for future clinical translation. Advanced delivery strategies, including nanoformulations, targeted delivery systems, and microbiota-based approaches, may enhance tissue exposure and optimize therapeutic outcomes. Finally, well-designed randomized controlled trials with standardized polyphenol preparations, pharmacokinetic evaluation, and clinically relevant behavioral and inflammatory biomarkers are urgently required to determine whether polyphenols can be effectively translated into dietary or therapeutic interventions for obesity-related inflammatory depression.

Overall, polyphenols represent promising candidates for modulating obesity-driven neuroinflammation and emotional dysfunction. Nevertheless, successful translation from experimental models to clinical applications will require a comprehensive understanding of molecular mechanisms, bioavailability limitations, individual metabolic differences, and long-term safety profiles.

## Figures and Tables

**Table 1:** Comparison of the present review with representative published reviews on related topics.

Review (Year)	Main Focus	Obesity as Primary Driver	Major Mechanisms Discussed	Dose Summary Provided	Advantage of the Present Review
[15]	Effects of obesity on depression via inflammation and gut microbiota	Yes	Gut microbiota dysbiosis, metabolic endotoxemia, BBB disruption, HPA axis, probiotics/prebiotics	No	Extends the mechanistic framework of obesity-related depression to include peripheral inflammation, dietary saturated fatty acids, and microglial lipid droplet accumulation
[16]	Polyphenols inhibiting MAPK pathway in depression	No	MAPK, NF-κB, PI3K/Akt pathways, oxidative stress	No	Focuses on obesity as the initiating metabolic stressor and explores the dose-dependent effects of polyphenols
[11]	Obesity as a threat to depression and anxiety prevalence	Yes	Adipose inflammation, gut dysbiosis, neuroinflammation, insulin/leptin resistance, HPA axis, neuroplasticity	No	Evaluates polyphenols as therapeutic agents and, for the first time, summarizes their effective dose ranges in both in vitro and in vivo obesity models
[17]	Polyphenols targeting neuroinflammation in inflammation-associated depression	No	NF-κB, NLRP3 inflammasome, monoamines, glutamate, HPA axis, neurogenesis	No	Links the actions of polyphenols to obesity-driven neuroinflammation, covering microglial lipid metabolism and saturated fatty acid accumulation-mechanisms that were not addressed in the study by Tayab et al.
[18]	Polyphenols as diet therapy in depressive disorders	No	Inflammation, oxidative stress, gut microbiota, HPA axis, neuroprotection	No	Provides a disease-specific mechanistic cascade from obesity to depression and includes a dose analysis of polyphenols
[19]	Shared biological mechanisms of depression and obesity	Yes	Adipokines, lipokines, inflammation, gut microbiota, neuroplasticity, HPA axis	No	Links obesity-driven metabolic stress with polyphenol-based interventions
[20]	Plant polyphenol extracts treating depression via anti-neuroinflammation	No	Microglia, astrocytes, NF-κB, MAPK, PI3K/Akt, NLRP3	No	Focuses on obesity as the initiating metabolic stressor and explores the dose-dependent effects of polyphenols
[21]	Phytotherapy in overweight/obesity comorbid with depressive symptoms	Yes	AMPK, BDNF, NF-κB, gut–brain axis, inflammation, oxidative stress	No	Offers greater mechanistic depth in neuroinflammatory pathways (e.g., microglial lipid droplets and SFA-induced TLR4 signaling) and provides a more detailed dose summary

**Table 2:** (**A**) Effective doses and mechanisms of polyphenols for ameliorating obesity-related neuroinflammation in in vitro. (**B**) Effective doses and mechanisms of polyphenols for ameliorating obesity-related neuroinflammation in in vivo.

(**A**)						
**Compound**	**Cell Model**		**Intervention Protocol (Effective Doses and Treatment Duration)**	**Therapeutic Effects**	**Main Mechanisms**	**References**
EGCG	BV-2 cell 1 µg/mL LPS (1 h)		150 µM, 24 h	suppress inflammatory responses	↓ mTOR, NF-κB, STAT1, Akt, CCL5, SMAD3; ↑ Ins2, Pld2, A20/TNFAIP3, GAB1	[93]
Baicalin	BV-2 cell 0.1 µg/mL LPS (12 h)		7.5, 22.5 µM, 1 h	attenuate neuroinflammation	↓ TLR4/MyD88/NF-κB, MAPK, miR-55	[80]
Baicalein	BV-2 cell 0.1 µg/mL LPS (24 h)		45 µM, 24 h	inhibit pro-inflammatory microglial polarization	↓ TLR4, NF-kB, STAT1	[99]
Punicalagin	BV-2 cell 1 µg/mL LPS (24 h)		25–100 µM, 30 min	anti-inflammatory and antioxidant	↓ p-ERK, p-JNK, p-STAT3, MAPK/NF-kB, NLRP3, iNOS, COX-2	[83]
Hispidulin	BV-2 cell 0.1 µg/mL LPS (24 h)		3–30 µM, 1 h	attenuate neuroinflammation	↓ NF-κB, STAT3, Akt, ROS, NO	[100]
Sinapic acid	BV-2 cell 0.1 µg/mL LPS (12 h)		10–20 µM, 1 h	attenuate neuroinflammation	↓ Akt, MAPK, NO, IL-6	[79]
Hesperetin	BV-2 cell 1 µg/mL LPS (24 h)		25–100 µM, 24 h	anti-inflammatory and antioxidant	↓ Keap1/Nrf2, NF-kB; ↑ HMOX1, GCLC	[73]
Açaí extract	BV-2 cell 1 µg/mL LPS (72 h)		0.001–10 µg/mL, 72 h	attenuate neuroinflammation	↓ ROS, IL-1β, IL-6, TNF-α	[101]
Magnolol	BV-2 cell LPS/ATP		10–20 µM, 2 h	promote anti-inflammatory microglial polarization	↓ NLRP3, caspase-1, IL-1β; ↑Nrf2, HO-1	[102]
Lychee seed polyphenol	BV-2 cell Aβ(1–42) peptide		5, 10 µg/mL, 24 h	regulate autophagy and inhibit inflammation	↓ NLRP3, ASC, caspase-1, IL-1β	[103]
Punicalin	BV-2 cell 0.1 µg/mL LPS (24 h)		1, 5, 10 µg/mL, 24 h	attenuate neuroinflammation	↓ TLR4, NF-kB, IL-1β, IL-6, TNF-α	[104]
(**B**)						
**Compound**	**Animal Model**	**Intervention Protocol (Effective Doses and Treatment Duration)**	**Therapeutic Effects**	**Main Mechanisms**	**Bioavailability/Translational Considerations**	**References**
Apple polyphenol	high-fat/cholesterol diet-induced obesity mice	gavage, 125 and 500 mg/(kg.bw.d), 8 weeks	ameliorate obesity and neuroinflammation	blood–brain barrier protection: ↓NF-κB, MyD88, TRIF, IKKβ; ↑ Occludin, ZO-1	low bioavailability; human equivalent dose exceeds reasonable range	[105]
Apple polyphenol	high-sugary diet-induced depressed mice	gavage, 500 mg/(kg.bw.d), 8 weeks	ameliorate obesity, gut microbiota, neuroinflammation, and depressive-like behaviors	gut–brain axis: ↓ NF-κB, GC, ACTH; ↑ IL-10, MUC-2, Occludin, ZO-1	low bioavailability; human equivalent dose exceeds reasonable range	[71]
Isorhamnetin	high-fat and high-fructose diet-induced obesity mice	0.03% and 0.06% *w*/*w*, mixed in diet, 14 weeks	ameliorate obesity and neuroinflammation	inhibition of microglial activation: ↓ p-JNK, p-p38, p-NF-κB, IL-1β, TNF-α; ↑ CREB/BDNF	BBB penetrable; brain concentration data limiting	[82]
Genistein	diabetes-induced brain damage mice	600 mg/kg, mixed in diet, 4 weeks	ameliorate obesity, neuroinflammation, and insulin resistance	↑ p-Akt, NGF, BDNF	BBB penetrable; brain concentration data limiting	[87]
EC	high-fat diet-induced depressed mice	gavage, 2 and 20 mg/(kg.bw.d), 24 weeks	o effect on obesity; improves gut microbiota and depressive-like behaviors	gut–brain axis modulation; ↑ Lactobacillus, Enterobacter; ↑ BDNF, GR, MR; ↓ 11β-HSD1	high bioavailability; BBB penetrable; human data limited	[96]
EGCG	high-fat diet-induced obesity mice	gavage, 25, 50, and 100 mg/(kg.bw.d), 6 weeks	ameliorate obesity, gut microbiota, neuroinflammation	Gut–brain axis modulation; ↑ Muribaculaceae, Alloprevotella; ↓ SCFAs, PPAR-γ, NF-ĸB	low bioavailability	[95]
Sea-buckthorn flavonoids	high-fructose diet-induced obesity mice	0.06% and 0.31% *w*/*w*, mixed in diet, 14 weeks	ameliorate obesity, neuroinflammation, and insulin resistance	insulin resistance modulation: ↑ IRS-1/AKT, ERK/CREB/BDNF, PSD-95; ↓ NF-kB, IL-1β, iNOS, COX-2	bioavailability 26.3% in rodents (intestinal release); human data limited	[90]
Puerarin	high-fat diet-induced depressed mice	gavage, 150 mg/kg/day, 6 weeks	ameliorate neural plasticity and depressive-like behaviors	↓ GC, IL-1β; ↑ GLP-1R/BDNF/TrkB, 5-HT	BBB penetrable; brain concentration data limiting	[106]
Theabrownin	high-fat diet-induced brain damage mice	gavage, 180 and 360 mg/kg/day, 8 weeks	ameliorate obesity, neuroinflammation, and hippocampal damage	↓ MARK4/NLRP3, BAX; ↑ PSD95, SYN1, SYP, Bcl-2	Macromolecular; low bioavailability	[107]
Hydroxytyrosol	High-fat and high-fructose diet-induced cognitive impairment mice	gavage, 25, 50 and 100 mg/kg/day, 14 weeks	ameliorate obesity, neuroinflammation, and hippocampal damage	↓ IL-1β, IL-6, TNF-α; ↑ PSD95, SNAP-25, BDNF	BBB penetrable; potential as a dietary supplement; human data limited	[108]

## Data Availability

No new data were created or analyzed in this study. Data sharing is not applicable to this article.

## Abbreviations

AChE: acetylcholinesterase; Akt: protein Kinase B; BBB: blood–brain barrier; BDNF: brain-derived neurotrophic factor; CNS: central nervous system; CRP: C-reactive protein; DA: dopamine; HFD: high-fat diet; HFFD: high-fat and high-fructose diet; HPA: hypothalamic–pituitary–adrenal; 5-HT: serotonin; IĸB: inhibitory ĸB protein; IKK: IĸB kinase; IL-1β: interleukin-1β; IL-6: interleukin-6; iNOS: inducible nitric oxide synthase; InsR: insulin receptor; IRS: insulin receptor substrate; JNK: c-Jun N-terminal kinase; LDs: lipid droplets; LepR: leptin receptor; LPS: lipopolysaccharide; MAO-A: monoamine oxidase-A; MAPK: mitogen-activated protein kinase; NE: norepinephrine; NF-ĸB: nuclear factor kappa-B; NLRP3: nod-like receptor family pyrin domain containing 3; PA: palmitic acid; PI3K: phosphatidylinositol-3-kinase; SFA: saturated fatty acids; STAT3: signal transducer and activator of transcription 3; TLR4: Toll-like receptor 4; TNF-α: tumor necrosis factor-α; WHO: World Health Organization; ZO-1: zonula occludens-1.
